# Highly Sensitive Detection of Bacteria by Binder-Coupled Multifunctional Polymeric Dyes

**DOI:** 10.3390/polym15122723

**Published:** 2023-06-18

**Authors:** Kriti Kapil, Shirley Xu, Inseon Lee, Hironobu Murata, Seok-Joon Kwon, Jonathan S. Dordick, Krzysztof Matyjaszewski

**Affiliations:** 1 Department of Chemistry, Carnegie Mellon University, 4400 Fifth Avenue, Pittsburgh, PA 15213, USA; kkapil@andrew.cmu.edu (K.K.); hiromura@andrew.cmu.edu (H.M.); 2 Department of Chemical and Biological Engineering, Center for Biotechnology & Interdisciplinary Studies, Rensselaer Polytechnic Institute, Troy, NY 12180, USA; xus11@rpi.edu (S.X.); leei5@rpi.edu (I.L.); kwons2@rpi.edu (S.-J.K.)

**Keywords:** pathogen identification, bioimaging, fluorescence, copolymer, ATRP, flow cytometry, confocal imaging

## Abstract

Infectious diseases caused by pathogens are a health burden, but traditional pathogen identification methods are complex and time-consuming. In this work, we have developed well-defined, multifunctional copolymers with rhodamine B dye synthesized by atom transfer radical polymerization (ATRP) using fully oxygen-tolerant photoredox/copper dual catalysis. ATRP enabled the efficient synthesis of copolymers with multiple fluorescent dyes from a biotin-functionalized initiator. Biotinylated dye copolymers were conjugated to antibody (Ab) or cell-wall binding domain (CBD), resulting in a highly fluorescent polymeric dye-binder complex. We showed that the unique combination of multifunctional polymeric dyes and strain-specific Ab or CBD exhibited both enhanced fluorescence and target selectivity for bioimaging of *Staphylococcus aureus* by flow cytometry and confocal microscopy. The ATRP-derived polymeric dyes have the potential as biosensors for the detection of target DNA, protein, or bacteria, as well as bioimaging.

## 1. Introduction

Infectious diseases caused by pathogens such as bacteria, viruses, and fungi remain a great burden on humanity [1]. As an example, antimicrobial resistance (AMR) is a major threat to human health. AMR-related infections have killed as many people as AIDS (acquired immunodeficiency syndrome) or malaria [2]. Under these circumstances, target-specific identification of pathogens is critical for effective medical intervention and decontamination of the infected areas [3]. Colony counting, immunological, and polymerase chain reaction (PCR) techniques are the traditional methods for pathogen identification [4,5,6,7]. However, these methods require time-consuming and complicated procedures such as cell culture, antigen/antibody treatment, and cell lysis/DNA amplification [5,6,7]. In this context, fluorescent labeling and detection have emerged as promising tools for pathogen visualization and identification, due to their simple labeling procedure, sensitivity, and stability [3,8,9,10]. Moreover, conjugation of fluorescent materials with a biological binder such as an antibody [11,12], an aptamer [13,14], or the cell-wall binding domain of a lytic enzyme (CBD) [15,16,17] allows for the targeting of a specific pathogen.

Various classes of fluorescent materials such as small-molecule organic dyes [18,19,20,21], fluorescent proteins [22,23,24], self-fluorescent polymers [10,25,26,27], dye-labeled polymeric nanoparticles [28,29,30,31,32,33,34], and quantum dots [35,36,37,38] have been explored. Among these, fluorescent dyes have gained popularity due to their commercial availability, ease of operation, and high resolution [39]. This has paved the way for the development of fluorescent dye copolymers, which combine various physicochemical properties with optical emission. The copolymers are less prone to sequestration from cells and tissues and typically exhibit lower toxicity and better photostability than low-molecular-weight dyes [40], hence offering a simple yet effective approach for low-sensitivity, high-contrast imaging of bacterial cells.

The fluorescent labeling of block copolymers has been achieved through various methods, such as noncovalent encapsulation, direct labeling with fluorescent initiators or monomers, or post-polymerization modification [41]. A variety of dyes were used for fluorescent labeling, including carbocyanine dye (e.g., Cy5, Cy5.5, Cy7-azide), benzopyrylium dyes (e.g., DY-676 and DY-700), push–pull dyes (e.g., coumarins, Nile red), and xanthene dyes (e.g., rhodamine, fluorescein). Xanthene dyes are particularly attractive due to their brightness, high extinction coefficients, quantum yield, and exceptional chemical stability. Noncovalent encapsulation may lead to leakage of dye in biological media, resulting in high background signals and cytotoxicity [42,43]. Direct labeling enables the incorporation of fluorescent dye during the polymerization step [44]. The fluorescent initiator-bearing hydroxyl groups were used to initiate the ring-opening polymerization [45,46]. This approach limits the number of fluorescent markers per polymer chain [47,48]. Alternatively, the use of a fluorescent monomer allows for control over the dye content by controlling the number of fluorescent monomers incorporated during the polymerization. Before its incorporation, the fluorophore is converted to a monomer by functionalization with a polymerizable vinyl group [49]. Techniques such as free-radical polymerization [50,51,52,53,54,55,56], reversible addition–fragmentation chain transfer (RAFT) [57,58,59,60,61,62], atom transfer radical polymerization (ATRP) [63,64,65,66,67,68,69,70,71,72,73,74], and ring-opening metathesis polymerization (ROMP) [75,76] have been used to incorporate fluorophores into block copolymers.

Despite the long history of employing polymers for bioanalytical applications, high-dispersity polymers generally offer limited control over functionality and topology [77,78,79]. ATRP has emerged as one of the most versatile and powerful reversible deactivation radical polymerization (RDRP) techniques, offering precise control over molecular weight, molecular weight distribution, functionality, architecture as well as tolerance to most functional groups [80,81,82,83,84,85,86,87,88,89,90,91,92,93,94]. ATRP is characterized by an equilibrium established through an inner-sphere electron-transfer process mediated by a transition metal complex, usually the activator [Cu(I)-L]^+^ (L typically being a polydentate amine ligand), which reacts with an alkyl halide initiator (R-X), leading to the formation of a [X–Cu(II)/L]^+^ deactivator and a propagating radical (R*). Radical propagation occurs until the radical chain ends are deactivated by [X–Cu (II)/L]^+^, forming X-capped dormant species and regenerating [Cu(I)-L]^+^. ATRP equilibrium is shifted toward dormant species since the rate constant of activation of dormant species is typically much smaller than the rate constant of radical deactivation, i.e., k_act_ ≪ k_deact_. Thus, the key aspect of the ATRP mechanism is a low concentration of active propagating species and a larger number of dormant chains [81,95,96,97,98]. Over the years, the scope of ATRP has been expanded to various solvents and reaction conditions, including water at room temperature using a low concentration of copper catalyst and no protective atmosphere of inert gas [99,100,101,102,103,104]. By optimizing the polymerization conditions and parameters, the copolymerization kinetics can be controlled.

Over the last decade, photoinduced ATRP techniques have been developed to harness the power of light to generate radicals [85,105,106]. Recently, photoinduced ATRP using copper complexes to achieve controlled radical propagation and photocatalyst to trigger and drive polymerization has been explored [107]. Our group reported green-light-induced ATRP with dual catalysis, using eosin Y (EYH_2_) in combination with a copper complex as a highly efficient method for rapid and well-controlled polymerization of oligo (ethylene oxide) methyl ether methacrylate [108] and oligo (ethylene oxide) methyl ether acrylate [109] in water under ambient conditions without the need for deoxygenation. The scope of the technique has been demonstrated by controlled polymerization of a variety of monomers, hyperbranched polymers with a tunable degree of branching [110] and grafting from the surface of biomolecules to synthesize well-defined protein–polymer [97,105,111,112,113,114,115] and DNA–polymer bioconjugates [97,116,117]. 

Herein, we report the synthesis and characterization of a polymeric dye complex containing a binder such as an antibody or the cell-wall binding domain (CBD) from a lytic enzyme for a highly sensitive bioimaging technique for pathogen identification. Putative autolysin from *Staphylococcus aureus* (SA1) contains a cysteine, histidine-dependent amidohydrolases/peptidases (CHAP) domain and a putative CBD [118]. The CBD from SA1 was successfully expressed and exhibited high selectivity to *Staphylococcus aureus*, similar to the CBD of lysostaphin, a lytic enzyme known for its specific targeting of *Staphylococcus aureus* [119]. The approach involves the coupling of *Staphylococcus aureus* targeting polyclonal antibody or CBD of SA1 to rhodamine dye-labeled copolymers comprised of oligo (ethylene oxide) methyl ether methacrylate or carboxy betaine methacrylate (CBMA). The polymers were grafted from a biotin-functionalized ATRP initiator under blue-light irradiation using Eosin-Y/Cu-mediated fully oxygen-tolerant ATRP technique. The nondestructive binding properties of the copolymeric dye complex were tested on the target bacterial cells and were applied to the bioimaging of target bacteria using fluorescence detection and confocal microscopy analysis.

## 2. Materials and Methods

### 2.1. Materials

All chemicals were purchased from commercial sources and used as received unless otherwise noted. Tris(2-pyridylmethyl) amine (TPMA, 99%) was purchased from AmBeed (Arlington Heights, IL, USA). Methacryloxyethyl thiocarbamoyl rhodamine B (RDMA) was purchased from Polysciences (Warrington, PA, USA). 3-[[2-(Methacryloyloxy)ethyl] dimethylammonio] propionate (CBMA) was purchased from TCI (Tokyo, Japan). Water (HPLC grade), dimethylformamide (DMF, ≥99.8%), and dimethyl sulfoxide (DMSO, ≥99.7%) were purchased from Fisher (Waltham, MA, USA). *Staphylococcus aureus* (ATCC 6538) (*S. aureus*) and *Bacillus anthracis* Sterne (*B. anthracis*) were purchased from the American Type Culture Collection (ATCC) (Manassas, VA, USA). Polyclonal antibody against *Staphylococcus aureus* was purchased from Invitrogen (Waltham, MA, USA). Eosin Y (EYH_2_, 99%), copper (II) bromide (CuBr_2_, 99.99%), triethanolamine (TEOA, ≥99.0%), and NeutrAvidin were purchased from Thermo Fisher Scientific (Waltham, MA, USA). Biotinylated rhodamine B was purchased from Nanocs (New York, NY, USA). Luria–Bertani (LB) medium and agar were purchased from Becton Dickinson (Franklin Lakes, NJ, USA). BL21(DE3) competent cells and restriction enzymes such as NdeI and XhoI were purchased from New England Biolabs (NEB) (Ipswich, MA, USA). Oligo (ethylene oxide) methyl ether methacrylate (average M_n_ = 500, OEOMA_500_), 1,4-bis(3-isocyanopropyl) piperazine (QA), ampicillin, isopropyl-β-d-thiogalactoside (IPTG), deoxyribonuclease (Dnase) I from bovine pancreas, phenylmethanesulfonylfluoride (PMSF), Tween20, glycerol, imidazole, phosphate-buffered saline (PBS), D-biotin, paraformaldehyde (PFA), sodium phosphate, sodium hydroxide (NaOH), and sodium chloride (NaCl) were purchased from Sigma Aldrich (St. Louis, MO, USA). Lysozyme and nickel-NTA agarose beads were purchased from Gold Biotechnology (St. Louis, MO, USA). All solutions were prepared with purified water by a Milli-Q purification system from Millipore (Burlington, MA, USA). The biotinylated ATRP initiator (Biotin-I) was synthesized according to a previously reported procedure [110].

### 2.2. Synthesis of Biotinylated Dye Copolymers

OEOMA_500_ was passed through a column of basic alumina to remove the inhibitor. The stock solutions of RDMA (20 mg in 2.0 mL of DMSO), biotin-I (20 mg in 1.0 mL of DMSO), CuBr_2_ (33.5 mg in 20.0 mL of DMSO), TPMA (13.06 mg in 1.0 mL of DMSO), and EYH_2_ (0.97 mg in 1.0 mL of DMSO) were prepared prior to polymerization. 

BT-p(CBMA-RDMA (2)): In a 5 mL volumetric flask, 344 mg (1.5 mmol) of CBMA were weighed. CuBr_2_ stock (200 µL), TPMA stock (100 µL), biotin-I (200 µL), EYH_2_ stock (50 µL), RDMA stock (1 mL), DMF (50 µL), and 10X PBS solution (500 µL) were added. Finally, HPLC water was added to reach a final volume of 5 mL, and the reaction mixture was stirred on a vortex. The final concentrations were CBMA (300 mM), RDMA (3 mM), biotin-I (1.5 mM), EYH_2_ (15 µM), CuBr_2_ (0.3 mM), TPMA (0.9 mM), and DMSO (30% *v*/*v*). ([CBMA]/[RDMA]/[biotin-I]/[EYH_2_]/[CuBr_2_]/[TPMA] = 200/2/1/0.02/0.4/1.2). Then, 4.4 mL of the reaction “cocktail” were added to a 1-dram (12/96 mm) vial equipped with a magnetic stirring bar. The polymerization mixture was stirred in an open vial at 500 rpm for 60 min under blue LEDs (450 nm, 25.0 mW/cm^2^).

BT-p(OEOMA_500_-RDMA (2/4): In a 5 mL volumetric flask, 750 mg (1.5 mmol) of OEOMA_500_ were weighed. CuBr_2_ stock (200 µL), TPMA stock (100 µL), biotin-I (100 µL), EYH_2_ stock (50 µL), RDMA stock (500 µL), DMF (50 µL), and 10X PBS solution (500 µL) were added. Finally, HPLC water was added to reach a final volume of 5 mL, and the reaction mixture was stirred on a vortex. The final concentrations were OEOMA_500_ (300 mM), RDMA (1.5 mM), biotin-I (0.75 mM), EYH_2_ (15 µM), CuBr_2_ (0.3 mM), TPMA (0.9 mM), and DMSO (30% *v*/*v*). ([OEOMA_500_]/[RDMA]/[biotin-I]/[EYH_2_]/[CuBr_2_]/[TPMA] = 400/2/1/0.02/0.4/1.2). For the synthesis of BT-p(OEOMA_500_-RDMA (4)), RDMA stock (1.0 mL) was added, resulting in the final concentration of RDMA (3 mM) in the polymerization mixture. ([OEOMA_500_]/[RDMA]/[biotin-I]/[EYH_2_]/[CuBr_2_]/[TPMA] = 400/4/1/0.02/0.4/1.2). Then, 4.4 mL of the CRBP cocktail were added to a 1-dram (12/96 mm) vial equipped with a magnetic stirring bar. The polymerization mixture was stirred in an open vial at 500 rpm for 60 min under blue LEDs (450 nm, 25.0 mW/cm^2^).

### 2.3. Characterization of Biotinylated Copolymeric Rhodamine B by ^1^H NMR Spectroscopy and Size Exclusion Chromatography with Multi-Angle Light Scattering (SEC–MALS) Detectors

Before analysis, the synthesized biotinylated copolymeric dyes were purified by dialysis in deionized water using SpectraPor^®^ 10 kDA cutoff dialysis membrane for 48 h and then lyophilized. ^1^H NMR spectra were recorded on Bruker Avance III 500 MHz spectrometers with D_2_O as the solvent. SEC–MALS measurements were performed using the Agilent SEC system (Agilent, 1260 Infinity II with UV detector) coupled with MALS, DLS, Viscometer, and RI detectors (Wyatt Technology Corporation, Santa Barbara, CA, USA). Measurements were performed using the Waters Ultrahydrogel Linear column with 1X DPBS as an eluent at room temperature and a flow rate of 0.5 mL/min.

### 2.4. Kinetics of Photoinduced ATRP 

The ATRP reaction mixture (5 mL) was prepared according to the general procedure described above for the synthesis of BT-p(OEOMA_500_-RDMA (4)). The final concentrations were OEOMA_500_ (300 mM), RDMA (3.0 mM), biotin-I (0.75 mM), EYH_2_ (15 µM), CuBr_2_ (0.3 mM), TPMA (0.9 mM), and DMSO (30% *v*/*v*). ([OEOMA_500_]/[RDMA]/[biotin-I]/[EYH_2_]/[CuBr_2_]/[TPMA] = 400/4/1/0.02/0.4/1.2). Then, 4.4 mL of the ATRP cocktail ([OEOMA_500_]/[RDMA]/[biotin-I]/[EYH_2_]/[CuBr_2_]/[TPMA] = 400/4/1/0.02/0.4/1.2) were added to a 1-dram (12/96 mm) vial equipped with a magnetic stir bar. The polymerization mixture in an uncapped vial was stirred at 500 rpm for 40 min under green LEDs (520 nm, 9.0 mW/cm^2^). The samples were taken at regular intervals, quenched with 1,4-bis(3-isocyanopropyl) piperazine [120], and then analyzed by ^1^H NMR and SEC.

### 2.5. Photostability Assessment of Biotinylated Copolymeric Rhodamine B

The photostability of the biotinylated copolymeric dyes was accessed by measuring the fluorescence readout every 30 min for 18 h. The experiment was carried out overnight using a BioTek Synergy H1 microplate reader. The copolymer dye was dissolved in 1X PBS buffer to reach a final concentration of 60 μM and placed in a 96-well polystyrene black-bottom plate. Triplicate samples of the same concentration were measured with a control sample containing only the buffer. Mineral oil (50 μL) was added to each well to prevent evaporation. Samples were incubated in a plate reader (xenon flash lamp, high energy) at 37 °C in the measurement chamber. The fluorescence intensity was measured by top optics using the monochromator filter set: excitation at 540 nm and emission at 570 nm, every 30 min. 

### 2.6. Preparation of Cell-Wall Binding Domain (CBD)

The DNA sequence of the cell-wall binding domain of SA1 [119] including an avi-tag and *BirA* (biotin ligase) was subcloned into a pGS-21a and pCDF-duet vector between NdeI and XhoI, respectively. BL21(DE3) competent cells were then cotransformed with pGS-21a with SA1BD gene and pCDF-duet with *birA* gene. One milliliter of the saturated overnight culture was inoculated into 100 mL of fresh LB media containing ampicillin (100 µg/mL) and grown until the absorbance at 600 nm reaches approximately 0.4. IPTG and D-biotin were added to a final concentration of 1 mM and 50 µM, respectively, and cells were cultured at 16 °C and 150 rpm overnight. Afterward, cells were pelleted by centrifuging (4000 rpm) at 4 °C for 15 min and resuspended in 10 mL of cell lysis buffer in native purification buffer (NPB, 20 mM sodium phosphate, and 500 mM NaCl, pH 8.0) containing PMSF (1 mM), lysozyme (100 µg/mL), bovine pancrease Dnase I (100 µg/mL), and glycerol (5%, *v*/*v*). The cell suspensions were sonicated in ice using Misonix Sonicator^®^ 3000 (Farmingdale, NY, USA) for 30 min with 1 s pulses and then centrifuged at 4000 rpm for 15 min to collect the supernatant. The His-tagged protein in the supernatant was purified using nickel nitrilotriacetic acid (Ni-NTA) affinity chromatography. The bound protein was washed once with NPB containing PMSF (1 mM) and five times with NPB containing imidazole (20 mM). The protein was eluted with an elution buffer (NPB containing imidazole (200 mM)) and was dialyzed against PBS at pH 7.4 using an 8 kDa molecular weight cutoff membrane (SpectrumLabs, Arden, NC, USA). Protein concentrations were determined spectrophotometrically at 280 nm using a NanoDrop ND-1000 (ThermoFisher, Waltham, MA, USA).

### 2.7. Construction and Characterization of Antibody/CBD-Copolymeric Rhodamine B Complex

To construct the antibody/CBD complexes with rhodamine B dye, biotinylated polyclonal antibody or CBD solution was first mixed with NeutrAvidin in phosphate-buffered saline (PBS, pH 7.4) and incubated at room temperature for 30 min. Biotinylated monomeric or copolymeric rhodamine B solution was then added to the mixture, followed by incubation at room temperature for 30 min. The molar ratio of biotinylated antibody/CBD, NeutrAvidin, and monomeric/copolymeric rhodamine B was 1:1:1.

Fluorescence from the prepared complexes in the presence of target bacteria were measured to determine the binding. Briefly, 30 µL of the saturated overnight culture were inoculated into 3 mL of fresh LB media and grown until the absorbance at 600 nm reached approximately 0.4. Afterward, cells were centrifuged and washed three times with PBS (pH 7.4). Antibody/CBD complex fusion proteins were added to the target cells (5 × 10^8^ cells/mL) and incubated at room temperature for 15 min. After incubation, the resulting mixtures were washed three times with PBS with 0.2% Tween20 to remove the unbound complex and adjusted to 5 × 10^8^ cells/mL. The fluorescence intensity of the mixture was then measured using a microplate reader (SpectraMax M5, San Jose, CA, USA) (λ_ex_ = 546 nm and λ_em_ = 580 nm). The fluorescence from complexes at the surface of bacteria was also measured using flow cytometry. Mixtures were diluted 10-fold in PBS to 10^7^ cells/mL prior to flow cytometry. Flow cytometry was performed using the BD LSRII flow cytometer (BD Biosciences, Franklin Lake, NJ, USA) with 20,000 events collected for each sample. Gating and further flow cytometry analysis were performed using FlowJo.

### 2.8. Confocal Laser Scanning Microscopy (CLSM)

Antibody/CBD complex fusion proteins were added to bacterial cells (5 × 10^8^ cells/mL) and incubated at room temperature for 15 min. After incubation, the resulting mixtures were washed three times with PBS with 0.2% Tween20 to remove the unbound complex and adjusted to 5 × 10^8^ cells/mL. A PFA solution (4%) was then added to the resulting mixtures to fix the bacteria cells. After incubation on ice for 30 min, the mixture was washed twice with PBS. Each 10 µL aliquot of the prepared mixed suspensions was added to clean glass slides and lightly covered using coverslips. The samples were excited at 546 nm, and the emission was recorded between 556 and 632 nm. The samples were examined by confocal laser scanning microscopy (LSM780) with a 93× glycerol immersion objective lens using a 546 nm laser (Carl Zeiss A.G., Oberkochen, Germany). Microscopy images were prepared and analyzed using ImageJ (Version 1.53k, National Institutes of Health, Bethesda, MD, USA). Briefly, raw TIF images for each microscopy sample were imported into ImageJ. For each image, the fluorescence was measured for 25 cells, and 5 background spots were measured for correction. The size of measurement for each cell and background was kept consistent within images. The corrected total cell fluorescence (CTCF) for each cell was calculated. Confocal microscopy was also used to examine the specificity of the antibody/CBD complex fusion proteins by using a mixture of both *S. aureus* and *B. anthracis* cells. The same procedure as above was used where the only modification is using a 1:1 mixture of *S. aureus* and *B. anthracis* cells. Confocal microscopy was performed using the same parameters above. Brightfield images for bacterial mixtures were also taken.

## 3. Results and Discussion

### 3.1. Synthesis and Characterization of Biotinylated Copolymeric Rhodamine B

**Figure 1:**
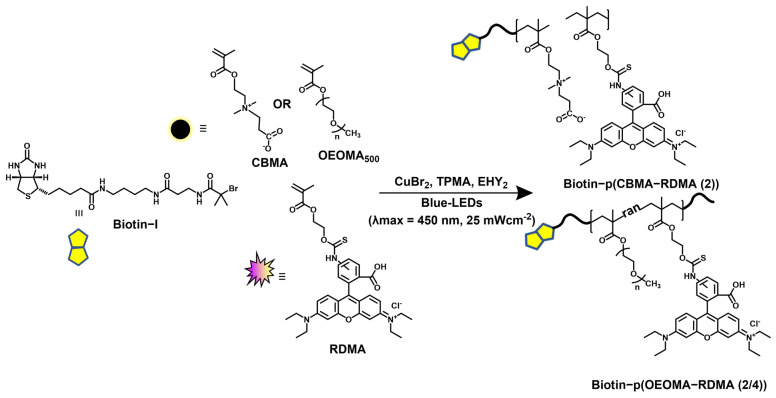
Synthesis of biotinylated multifunctional dye copolymers by photoredox/Cu-catalyzed, oxygen-tolerant ATRP.

The synthesis of biotinylated multifunctional dye copolymers was performed using a recently developed fully oxygen-tolerant, photoinduced atom transfer radical polymerization (ATRP) [108]. The copolymerization of zwitterionic and hydrophilic CBMA monomer and dye-labeled rhodamine-B-methacrylate (RDMA) monomer was performed in an aqueous medium under blue-light irradiation (λ_max_ = 450 nm, 25.0 mW/cm^2^), using biotin-I as the initiator, Eosin Y (EYH_2_) as the organic photoredox catalyst, and CuBr_2_/TPMA as the deactivator (Figure 1).

**Figure 2:**
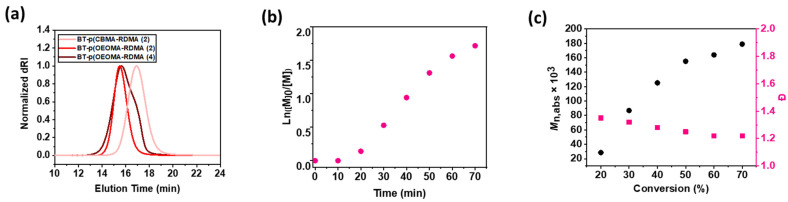
(**a**) SEC analysis of the biotinylated copolymer rhodamine dye in Table 1 after 60 min. Copolymerization kinetics of the optimized EYH_2_/Cu-catalyzed ATRP for the synthesis of BT-p(OEOMA_500_-RDMA (4)). (**b**) First-order kinetic plot. (**c**) Evolution of molecular weight and molecular weight distribution with conversion (black dots represent molecular weight and pink dots represent molecular weight distribution).

Similarly, amphiphilic copolymers comprised of PEG backbone were also synthesized by copolymerization of oligo (ethylene oxide) methyl ether methacrylate (OEOMA_500_) monomer with RDMA. ^1^H NMR was analyzed to compute conversion of monomers; the peak integral from 4.26 to 4.32 ppm corresponding to the monomer was set at 100 at t = 0, and the broad peak between 4.05 and 4.20 ppm corresponding to the polymer was observed at t = 60 min. Within 60 min, the CBMA and OEOMA_500,_ and RDMA reached a high conversion (≈80%), which was used to compute theoretical molecular weight (*M*_n,th_). ^1^H NMR spectra of the purified polymer samples was also recorded (Figures S1 and S2). The purified polymer samples were then analyzed by the SEC–MALS technique (Table 1), where a good agreement between *M*_n,th_ and the observed absolute molecular weight *(M*_n, abs_) revealed well-controlled polymerizations (Figure 2a).

The copolymerization kinetics of the photoinduced ATRP were performed under the optimized conditions ([OEOMA_500_]/[RDMA]/[biotin-I]/[EYH_2_]/[CuBr_2_]/[TPMA] = 400/4/1/0.02/0.4/1.2). The samples were taken at regular intervals, quenched with 1,4-bis(3-isocyanopropyl) piperazine, and monitored by ^1^H NMR in D_2_O. A short induction period (10 min) was followed by rapid polymerization, reaching 78% monomer conversion within 60 min (Figure 2b and Figure S3), and exhibited first-order kinetics. The monomer conversion determined via ^1^H NMR revealed statistical incorporation of the fluorescent monomer (RDMA) within the polymer chain. A good agreement between theoretical and experimental molecular weights was observed. In addition, SEC traces revealed that molecular weights increased as a function of monomer conversion, and dispersity values remained low *Ð* < 1.3). The ATRP technique enabled efficient and rapid synthesis of copolymer chains without the need for deoxygenation, with the desired degree of polymerization, desired molar ratio of RDMA, predictable molecular weights, narrow molecular weight distribution, and homogenously distributed fluorescent dye monomers (Figure 2c).

### 3.2. Characterization of Biotinylated Polymeric Dyes and Their Complexes with Selective Binders

**Figure 3:**
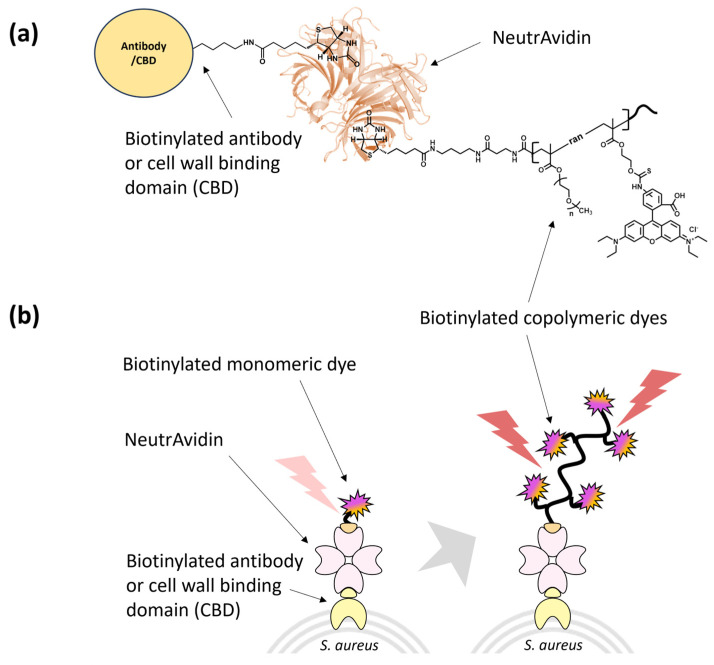
Schematic of (**a**) antibody/CBD-copolymeric rhodamine B complex, and (**b**) signal generation of antibody/CBD-monomeric and -copolymeric rhodamine B complexes on the target *S. aureus*.

**Figure 4:**
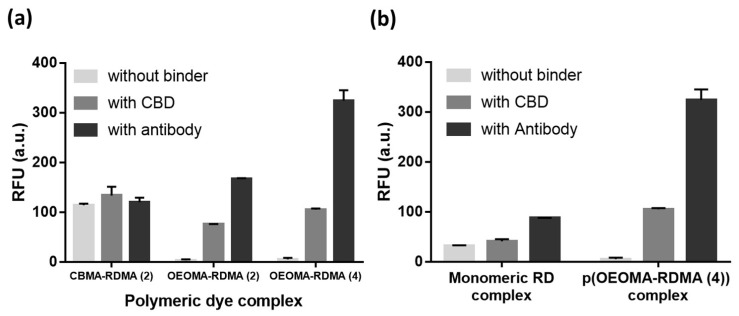
Comparison of fluorescence from CBD/antibody complex with (**a**) BT-p(CBMA-RDMA (2)), BT-p(OEOMA-RDMA (2)), and BT-p(OEOMA-RDMA (4)), and (**b**) BT-RD and BT-p(OEOMA_500_-RDMA (4)) at the surface of target bacteria.

We complexed the biotinylated polymeric dyes with NeutrAvidin and a cell binder such as biotinylated *S. aureus* polyclonal antibody or biotinylated CBD using biotin/NeutrAvidin interactions and tested their binding toward the target *S. aureus* cells using fluorescence detection (Figure 3). For fluorescence measurements of antibody/CBD-polymeric dye complexes on the surface of *S. aureus*, we used an excitation and emission at 546 nm and 580 nm, respectively, based on the fluorescent spectra of p(OEOMA500-RDMA (4)) (Figure S4). In both antibody and CBD cases, the p(OEOMA_500_-RDMA (4)) complex showed the best performance without any background fluorescence (Figure 4a) compared to p(OEOMA_500_-RDMA (2)) and p(CBMA-RDMA (2)) complexes. The result implies that a higher molar ratio of rhodamine B dye was incorporated in the p(OEOMA_500_-RDMA (4)) backbone than in the p(OEOMA_500_-RDMA (2)) backbone. Also, in the case of zwitterionic BT-p(CBMA-RDMA (2)), we observed a background fluorescence, which can be attributed to the distinct characteristics of the copolymer backbone, contributing to its zwitterionic and hydrophilic nature. This unique feature of the copolymer backbone may lead to nonspecific interactions to zwitterionic teichoic acid polymers located within the Gram-positive cell wall. [121,122], ^1^ (resulting in high background fluorescence. In contrast, both p(OEOMA_500_-RDMA (2)) and p(OEOMA_500_-RDMA (4)) complexes with PEGylated side chains can effectively minimize the nonspecific interactions with the cell surface. In all cases, the antibody-induced fluorescence was higher than the CBD-induced fluorescence. This may be because the number of binding sites for the polyclonal antibody is higher than that of CBD at the upper cell wall structure of the Gram-positive *S. aureus*. The concentration of p(OEOMA_500_-RDMA (4)) species in the complexation process was optimized and fixed at 6 µM (Figure S5).

To assess the stability of biotinylated copolymer rhodamine B, the fluorescence intensity of the synthesized copolymers was monitored overnight ([BT-p(OEOMA-RDMA (4)] = 60 μM). The polymer sample was incubated in 1X PBS buffer in a plate reader at 37 °C in the measurement chamber for 18 h. The negligible change in the fluorescence intensity of the polymer samples during the experiment indicated the high stability of the polymeric dyes under these experimental conditions (Figure S6).

Next, the fluorescence of antibody/CBD-p(OEOMA_500_-RDMA (4)) complexes was measured and compared with biotinylated monomeric rhodamine B complexes with NeutrAvidin and antibody/CBD (antibody/CBD-RD complexes) (Figure 3). The fluorescence of the antibody- and CBD-p(OEOMA_500_-RDMA (4)) complexes were 2.6 and 3.7 times higher than that of the antibody- and CBD-RD complexes (Figure 4b), respectively, suggesting that the signal of each binder-p(OEOMA_500_-RDMA (4)) complex was improved by increasing the number of rhodamine B dyes on the copolymer without background fluorescence from nonspecific binding of the copolymer. 

**Figure 5:**
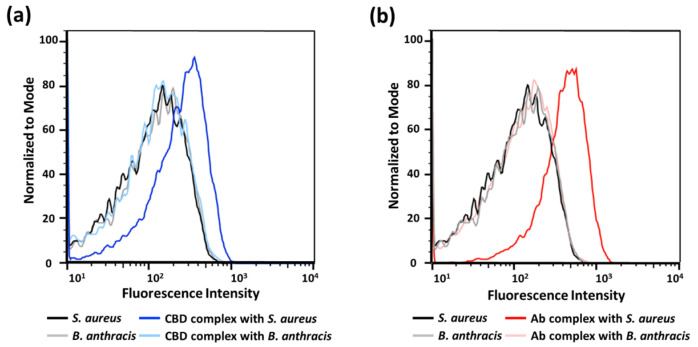
Flow cytometry analysis for (**a**) CBD-p(OEOMA_500_-RDMA (4)) complex and (**b**) antibody-p(OEOMA_500_-RDMA (4)) complex at the surface of bacteria.

We then assessed binding onto single bacterial cells using flow cytometry. The addition of each binder-p(OEOMA_500_-RDMA (4)) complex generated a clear change in the fluorescence intensity compared with the target *S. aureus* cells alone (Figure 5), suggesting that the fluorescence of each binder-p(OEOMA_500_-RDMA (4)) complex comes from binding to the surface of *S. aureus* cells. Furthermore, we confirmed the specificity of these complexes using flow cytometry. In both binder cases, the complex did not bind to *B. anthracis* Sterne cells, as no fluorescence was detected. These results suggest that we have generated complexes that are target-specific and can bind to the target bacterium, induced by antibody or CBD.

### 3.3. Antibody/CBD-Polymeric Dyes Complex for Bioimaging Application

**Figure 6:**
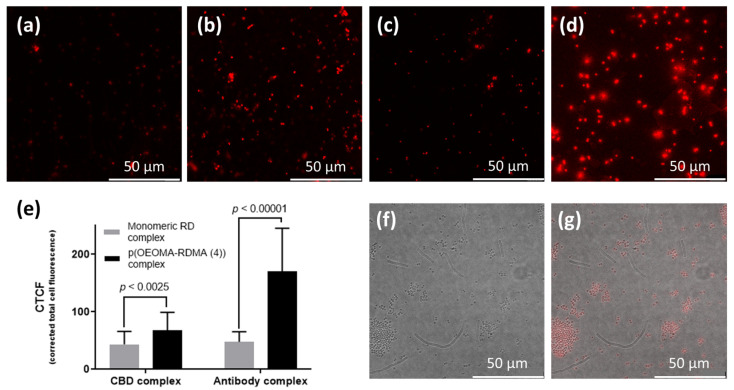
Confocal images of (**a**) CBD-monomeric RD and (**b**) CBD-p(OEOMA_500_-RDMA (4)), (**c**) antibody-monomeric RD, and (**d**) antibody-p(OEOMA_500_-RDMA (4)) complexes with *S. aureus* cells. (**e**) Comparison of corrected total cell fluorescence (CTCF) normalized by bacterial cell size in each confocal image (*n* = 15*). Confocal images for (**f**) fluorescent field, and (**g**) merged bright and fluorescent fields of antibody-p(OEOMA_500_-RDMA (4)) complex in the mixture of sphere-shaped *S. aureus* and the rod-shaped *B. anthracis* cells. The scale bar for all confocal images represents 10 µm.

We applied the signal-enhancing property of each binder-p(OEOMA_500_-RDMA (4)) complex for the bioimaging of target bacteria. When we prepared *S. aureus* cells with binder-p(OEOMA_500_-RDMA (4)) complex, all the target bacterial cells with binder-p(OEOMA_500_-RDMA (4)) complex showed red emission in each image (Figure 6a–d). In addition, the fluorescence images showed the same trends that we had previously obtained using fluorescence detection on a plate reader. Further analysis of these images was performed to quantify the fluorescence from the single cell by corrected total cell fluorescence (CTCF) analysis using the ImageJ program. The CTCF of antibody- and CBD-p(OEOMA_500_-RDMA (4)) complexes were 1.6 and 3.6 times higher than that of antibody- and CBD-RD complexes, respectively (Figure 6e). Additionally, in a mixture of cells, under confocal microscopy, we specifically distinguished the presence of the target bacteria from the nontarget using these complexes (Figure 6f,g). These results suggest that these binder-p(OEOMA_500_-RDMA (4)) complexes can be applied for bioimaging to visualize specific bacteria.

## 4. Conclusions

We have developed ATRP-derived copolymeric multifunctional rhodamine B dyes and attached them to binders such as an antibody or CBD for selective binding of target bacterial cells. The photoredox/Cu-catalyzed ATRP technique enabled the efficient and rapid synthesis of well-defined copolymers with multiple fluorescent dyes. Antibody/CBD-polymeric dye complex showed both enhanced fluorescence and target selectivity for bioimaging. This is due to the special structural property of this complex, consisting of multiple fluorescent dyes and a single binding molecule. The combination of this unique property of the polymeric dye and binder-induced targeting can also be applied to conjugate multiple signaling molecules such as quantum dots, DNA, and enzymes, followed by the potential applications in pathogen detection, and selective microbial decontamination, as well as bioimaging. The present work has also opened the potential application of ATRP-derived polymeric dyes in biosensors for detection of the DNA or protein biomarkers.

## Figures and Tables

**Table 1:** Synthesis of biotinylated multifunctional dye copolymers in water ^[a]^.

Entry	Sample Name	[M]/[Dye- M]/[I]	αM ^[b]^ (%)	*M*n,th ^[c]^	*M*n, abs ^[d]^	*Ð*
	Biotin-					
1	p(CBMA-	200/2/1	80%	36 600	40 200	1.25
	RDMA (2))					
	Biotin-					
2	p(OEOMA_500_-	400/2/1	84%	168 000	150 850	1.17
	RDMA (2))					
	Biotin-					
3	p(OEOMA_500_-	400/4/1	78%	158 600	149 200	1.23
	RDMA (4))					

^[a]^ Reactions conditions: [M] = 300 mM, [Dye-M] = [RDMA] = 1.5–3.0 mM, [I] = [Biotin-I] = 0.75–1.5 mM, [EYH_2_] = 15 M, [CuBr_2_] = 0.3 mM, [L] = [TPMA] = 0.9 mM in PBS with DMSO (30% *v*/*v*), irradiated for 60 min under blue LEDs (450 nm, 25.0 mW cm^2^), in an open vial with stirring at 500 rpm. Reaction volume 4.4 mL. ^[b]^ Monomer conversion was determined by using ^1^H NMR spectroscopy. ^[c]^ Theoretical molecular weight (*M*_n,th_) was calculated using the equation *M*_n,th_ = [M] × MW_M_ × α + MW-_Biotin-I_. ^[d]^ Absolute molecular weight (*M*_n, abs_) and *Ð* were determined by using SEC–MALS.

## Data Availability

Not applicable.

## Supplementary Materials

The following supporting information can be downloaded at: https://www.mdpi.com/article/10.3390/polym15122723/s1, Figure S1: ^1^H NMR spectrum of BT-p(CBMA-RDMA) in D_2_O; Figure S2: ^1^H NMR spectrum of BT-p(OEOMA-RDMA) in D_2_O; Figure S3: Overlapped ^1^H NMR spectra showing kinetics of EYH_2_/Cu-catalyzed blue-light-induced ATRP for copolymerization of OEOMA_500_ and RDMA; Figure S4: Fluorescence spectra of BT-p(OEOMA500-RDMA (4) (5 mg/mL) in PBS; Figure S5: Binding test using *S. aureus* cells with various concentrations of antibody/CBD-p(OEOMA_500_-RDMA (4)) complex; Figure S6: Evolution of the fluorescence intensity of BT-p(OEOMA_500_-RDMA (4)) (60 μM) overnight (37 °C, in PBS Buffer).
