# Supplementary material for: Highly Sensitive Detection of Bacteria by Binder-Coupled Multifunctional Polymeric Dyes
Source: Polymers (Basel). 2023 Jun 18;15(12):2723. doi: 10.3390/polym15122723 (PMC10304923; doi:10.3390/polym15122723)
Supplement: Supplementary file 1 [file polymers-15-02723-s001.zip › polymers-2440202-supplementary.pdf]

Supplementary Information

Highly sensitive detection of bacteria by the binder-coupled multifunctional polymeric dyes

Kriti Kapil [1,†], Shirley Xu [2,†], Inseon Lee [2], Hironobu Murata [1], Seok-Joon Kwon[2], Jonathan S. Dordick [2,*], and Krzysztof Matyjaszewski [1,*]

[1]Department of Chemistry, Carnegie Mellon University, 4400 Fifth Avenue, Pittsburgh, PA 15213, United States.

[2]Department of Chemical Engineering, Center for Biotechnology & Interdisciplinary Studies, Rensselaer Polytechnic Institute, Troy, NY 12180, United States.

* Correspondence: dordick@rpi.edu (J.S.D.); km3b@andrew.cmu.edu (K.M.)

† These authors contributed equally to this work

Supplementary Information

Synthesis & characterization of biotinylated copolymeric rhodamine B

The synthesis of biotinylated multifunctional dye-copolymers comprising of zwitterionic, or PEG-backbone was performed using a recently developed fully-oxygen tolerant, photo-catalyzed atom transfer radical polymerization (ATRP) described in the methods section 2.2 of the main text.

The biotinylated copolymer samples were purified by dialysis in deionized water using SpectraPor® 10 kDa cut-off dialysis membrane for 48 h in dark and then lyophilized. The purified samples were characterized by ^{1}H NMR spectra (Figures S1 and S2) and then by SEC-MALS (Figure 2a).

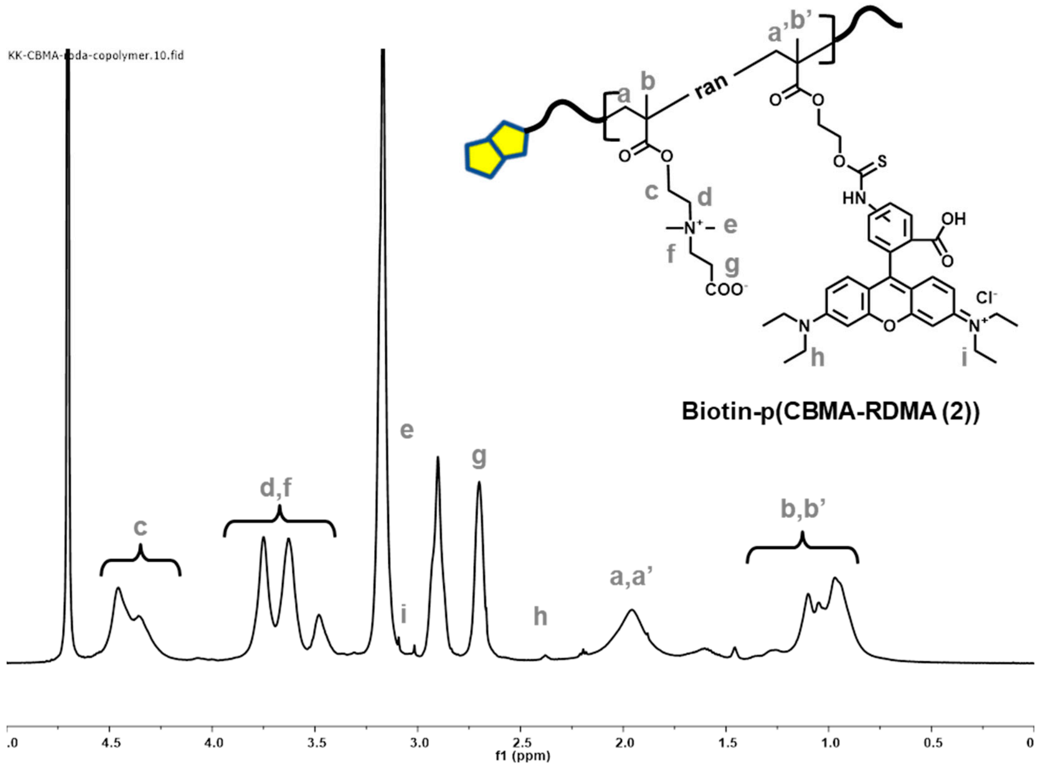

Figure S1. ^{1}H NMR spectrum of BT-p(CBMA-RDMA) in D_2O.

Supplementary Information

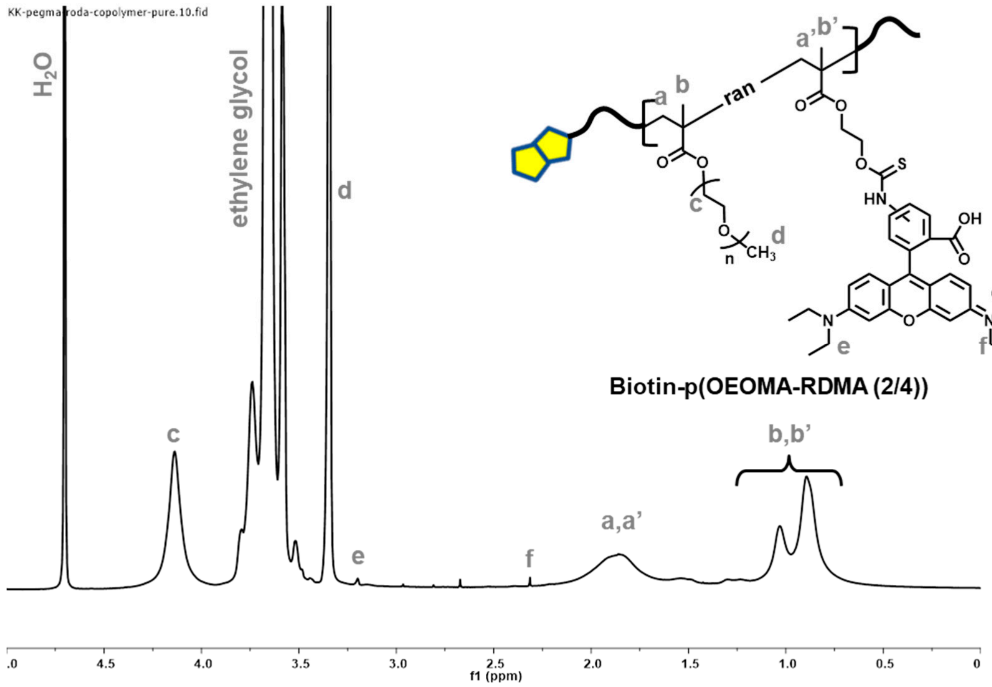

Figure S2. ^1H NMR spectrum of BT-p(OEOMA-RDMA) in D_2O.

Analysis of polymerization kinetics

The copolymerization kinetics of the photoinduced ATRP was performed under the optimized conditions ([$OEOMA_{500}$]/[RDMA]/[SBA]/[biotin-I]/[EYH_2]/[$CuBr_2$]/[TPMA] = 400/4/1/0.02/0.4/1.2). The samples were drawn at regular intervals and monitored by ^1H NMR in D_2O. A short induction period (10 min) was followed by the rapid polymerization reaching 78 % monomer conversion within 60 min (Figure S3).

Supplementary Information

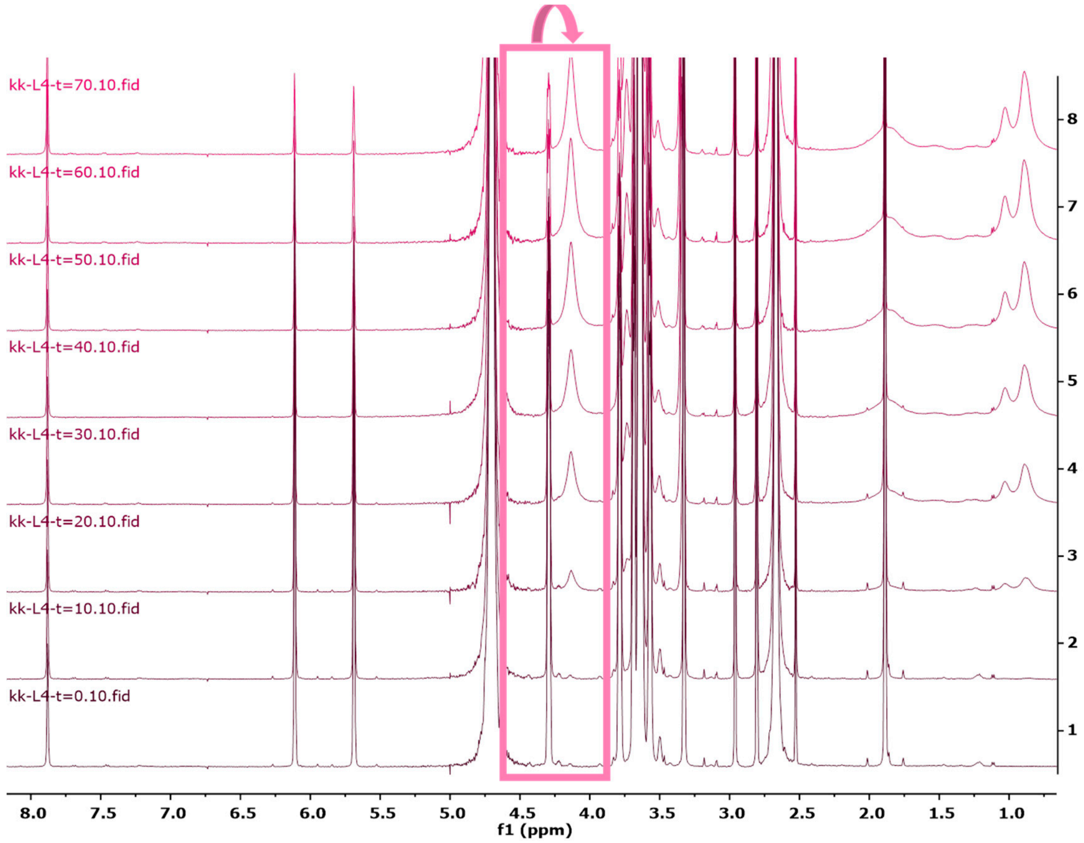

Figure S3: Overlapped ^{1}H NMR spectra showing kinetics of EYH_2/Cu-catalyzed blue-light induced ATRP for copolymerization of $OEOMA_{500}$ & RDMA.

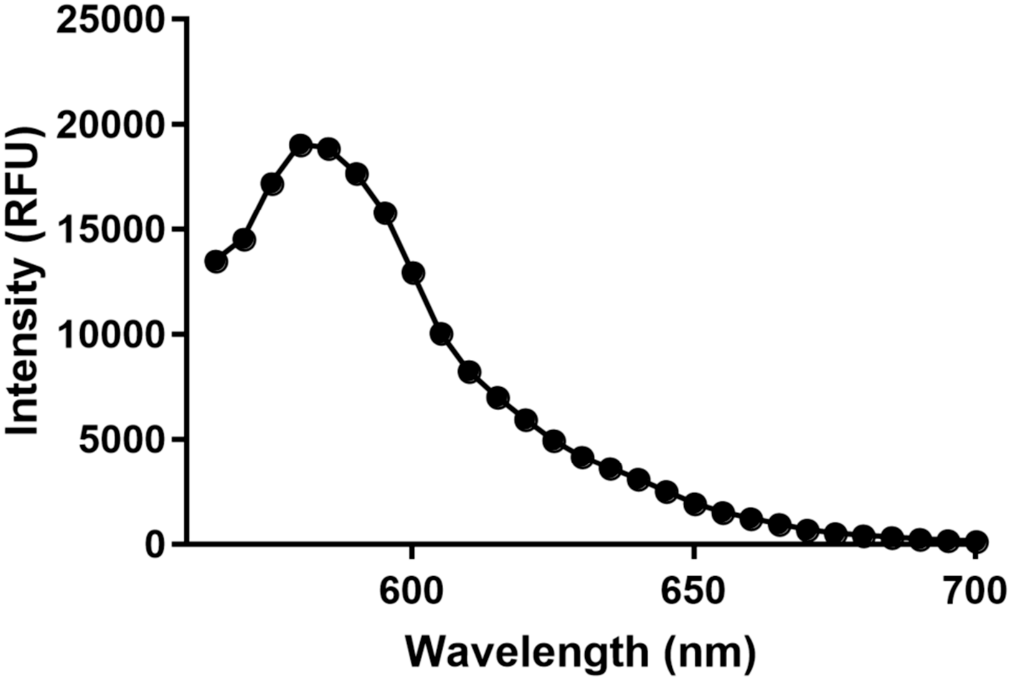

Figure S4. Fluorescence spectra of BT-p(OEOMA500-RDMA (4) (5 mg/mL) in PBS. Excitation wavelength is 546 nm.

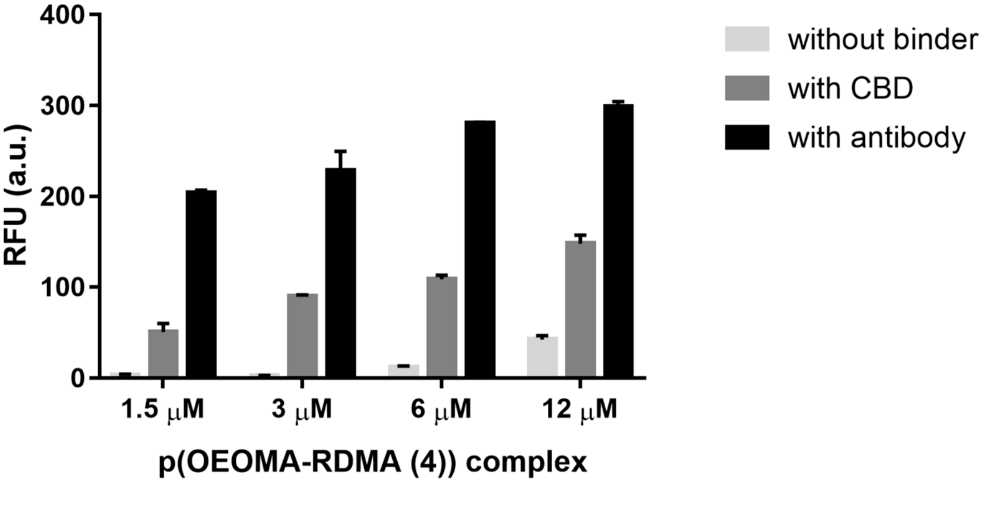

Figure S5. Binding test using *S. aureus* cells with various concentrations of antibody/CBD-p($OEOMA_{500}$-RDMA (4)) complex.

Supplementary Information

Photostability assessment of biotinylated copolymeric rhodamine B

To assess the stability of biotinylated copolymer rhodamine B, the fluorescence intensity of the synthesized copolymers was monitored overnight ([BT-p(OEOMA$_{500}$-RDMA (4)] = 60 µM) in a plate reader at 37 ^{0}C as described in section 2.5.

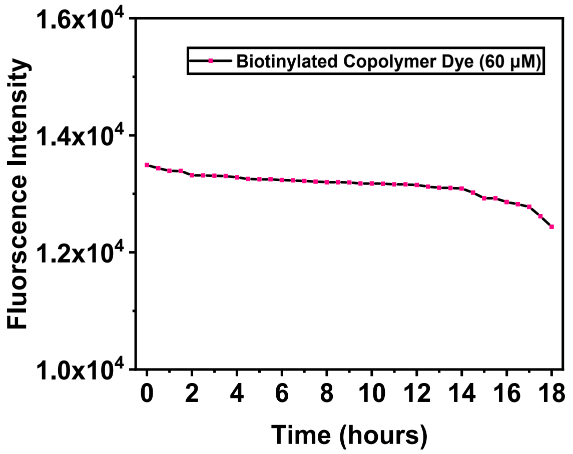

Figure S6. Evolution of the fluorescence intensity of BT-p(OEOMA$_{500}$-RDMA (4)) (60 µM) overnight (37 °C, in 1X PBS Buffer).
